# Predictive value of inflammatory prognostic index for contrast-induced nephropathy in patients undergoing coronary angiography and/or percutaneous coronary intervention

**DOI:** 10.1038/s41598-024-66880-7

**Published:** 2024-07-09

**Authors:** Yan Jiang, Baolin Luo, Yaqin Chen, Yanchun Peng, Wen Lu, Liangwan Chen, Yanjuan Lin

**Affiliations:** 1 Department of Nursing, Fujian Medical University Union Hospital, Fuzhou, Fujian China; 2 School of Nursing, Fujian Medical University, Fuzhou, Fujian China; 3 Department of Cardiovascular Surgery, Fujian Medical University Union Hospital, 29 Xinquan Road, Fuzhou, Fujian China; 4 Fujian Provincial Special Reserve Talents Laboratory, Fuzhou, Fujian China

**Keywords:** Inflammatory prognostic index, Prognosis, Coronary angiography, Percutaneous coronary intervention, Cardiovascular biology, Interventional cardiology, Myocardial infarction

## Abstract

The purpose of this study was to investigate the relationship between Inflammatory Prognostic Index (IPI) levels and Contrast-Induced Nephropathy (CIN) risk and postoperative clinical outcomes in patients undergoing coronary angiography (CAG) and/or percutaneous coronary intervention (PCI). A total of 3,340 consecutive patients who underwent CAG and/or PCI between May 2017 and December 2022 were enrolled in this study. Based on their baseline IPI levels, patients were categorized into four groups. Clinical characteristics and postoperative outcomes were compared among these groups. In-hospital outcomes focused on CIN risk, repeated revascularization, major bleeding, and major adverse cardiovascular events (MACEs), while the long-term outcome examined the all-cause readmission rate. Quartile analysis found a significant link between IPI levels and CIN risk, notably in the highest quartile (*P* < 0.001). Even after adjusting for baseline factors, this association remained significant, with an adjusted Odds Ratio (aOR) of 2.33 (95%CI 1.50–3.64; *P* = 0.001). Notably, baseline IPI level emerged as an independent predictor of severe arrhythmia, with aOR of 0.50 (95%CI 0.35–0.69; *P* < 0.001), particularly driven by the highest quartile. Furthermore, a significant correlation between IPI and acute myocardial infarction was observed (*P* < 0.001), which remained significant post-adjustment. For patients undergoing CAG and/or PCI, baseline IPI levels can independently predict clinical prognosis. As a comprehensive inflammation indicator, IPI effectively identifies high-risk patients post-procedure. This study underscores IPI's potential to assist medical professionals in making more precise clinical decisions, ultimately reducing mortality and readmission rates linked to cardiovascular disease (CVD).

## Introduction

Cardiovascular disease (CVD) is a prevalent chronic condition globally, with China experiencing a steady rise in its incidence, now estimated at 330 million patients annually^1^. Coronary heart disease (CHD) is particularly prevalent and associated with high mortality rates^2^. Consequently, coronary angiography (CAG) and percutaneous coronary intervention (PCI) are crucial diagnostic and treatment modalities. However, despite PCI's benefits for long-term prognosis, risks like contrast-induced nephropathy and major adverse cardiovascular events (MACE) remain significant^3,4^, contributing to increased mortality and readmission rates.

Contrast-induced nephropathy (CIN) refers to acute kidney injury following exposure to iodinated contrast agents. It typically occurs 1–3 days post-exposure, associated with poorer prognosis including increased hospitalization rates and costs^4,6^. Incidence can range from 20 to 25% in patients undergoing CAG and/or PCI, reaching 40% in high-risk cohorts^7,8^. While the exact cause of CIN variability remains unclear, enhanced inflammatory response is considered significant. Identifying inflammatory markers predicting CIN is crucial to mitigate complications. Neutrophil–lymphocyte ratio (NLR), C-reactive protein (CRP), and serum albumin (ALB) are established indicators of inflammation and atherosclerosis pathogenesis. Combining these into an inflammatory prognostic index (IPI) offers a more comprehensive assessment of inflammatory status. IPI has shown clinical significance in various fields but lacks evidence in predicting CIN and its relationship with clinical outcomes in CAG and/or PCI patients is unclear.

At present, many new biomedical research tools have been developed in the medical field to predict the association between metabolites and diseases^40–42,46^. However, the use of new research tools is more complex, and simple biomarkers are urgently needed. Some simple detection techniques have emerged^48^, but because hematological biomarkers are the easiest to obtain, they have received the attention of scholars and experts. NLR^9^, CRP^10^, and ALB^11^ indicate systemic inflammation and atherosclerosis development. However, individual markers lack completeness. Building upon this rationale, some scholars have proposed an IPI^12^. IPI combines CRP, NLR, and ALB, offering a better reflection of patients' inflammatory status. Initially used in predicting outcomes for cancer patients^13^, its significance in cardiovascular contexts, like cardiac surgery^14^, has been recognized. Yet, its predictive value for CIN in CAG and/or PCI patients and its link to clinical outcomes remain unexplored.

This study aims to investigate the role of IPI in predicting CIN risk and clinical outcomes in patients undergoing CAG and/or PCI, offering a practical predictive method. This could assist healthcare professionals in making more precise decisions, potentially lowering mortality and readmission rates in CHD patients.

## Methods

### Study design and population

**Figure 1:**
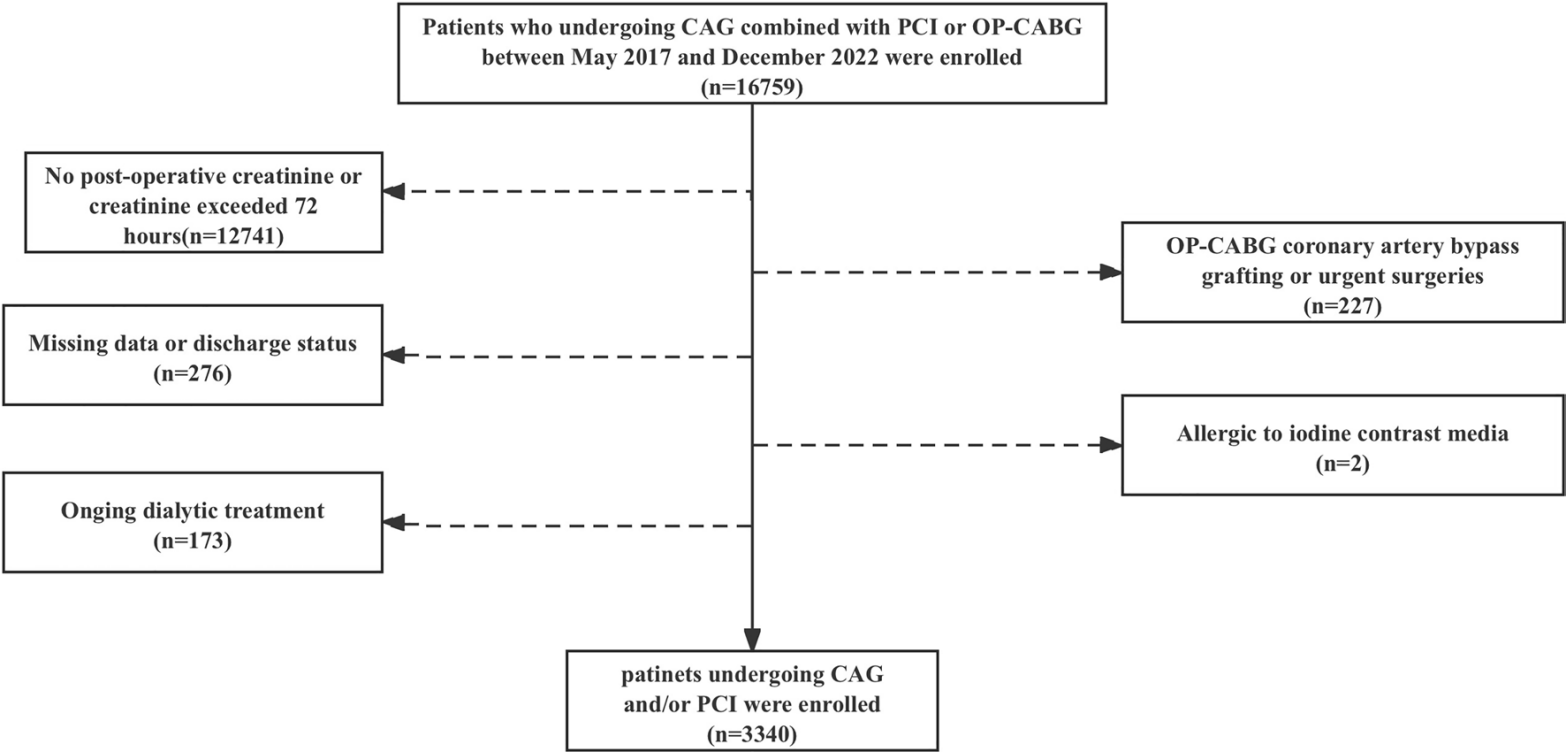
Inclusion/exclusion flowchart for the study group. Abbreviations: CAG, coronary angiography; PCI, percutaneous coronary intervention; OP-CABG, on-pump coronary artery bypass grafting.

We collected retrospective data from 16,759 patients undergoing CAG and/or PCI at Fujian Medical University Union Hospital between May 2017 and December 2022. Exclusion criteria were age < 18, missing or limited serum creatinine data, on-pump coronary artery bypass grafting (CABG), contrast agent allergies, ongoing dialysis, and missing data or discharge status. 3,340 patients were included for analysis. Inclusion/exclusion flowchart for the study groupis shown in Fig. 1. The study was approved by the hospital's Ethics Committee and followed the Declaration of Helsinki.

### Data collection and clinical definition

We obtained baseline data from the Electronic Medical Records System of Fujian Medical University Union Hospital, which primarily comprised sociodemographic information, admission and discharge diagnoses, results of laboratory and imaging tests, medication details, procedural characteristics, and discharge outcomes. Laboratory tests were conducted at the Laboratory Center of Fujian Medical University Union Hospital. Venous blood samples were collected after a fasting period of over 8 h, with water deprivation during the final 3–4 h of the fast.

Hypertension, diabetes mellitus (DM) and stroke were defined using the 10th Revision Codes of the International Classification of Diseases (ICD-10)^15^. The guidelines for the prevention and treatment of dyslipidemia in Chinese adults (2016)^16^, were used to define the diagnosis of hyperlipemia. The diagnostic criteria of chronic kidney disease (CKD) were based on Guidelines for the Screening, Diagnosis and Prevention of Chronic kidney Disease in China (2017)^17^, estimated glomerular filtration rate (eGFR) < 60 mL/min/1.73m^2^ and calculated with the modification of diet in renal disease (MDRD) formula^18^. CIN was defined as an absolute increase 44.2 μmol/L (0.5 mg/dL) or a relative increase of 25% in serum creatinine level from baseline within 48 to 72 h after intravascular use of iodinated contrast agents.

### Measurements

Venous blood samples collected within 24 h of admission were used to measure neutrophils (N), lymphocytes (L), ALB, and CRP. These parameters were used to calculate two inflammatory biomarkers: the neutrophil-to-lymphocyte ratio (NLR = N/L) and the inflammatory prognostic index (IPI = CRP × NLR/ALB). Baseline values for other laboratory parameters were determined based on recent preoperative serum creatinine levels and additional indicators.

### Outcomes measured

In-hospital outcomes included CIN risk, repeated revascularization, major bleeding, and MACE. Repeated revascularization meant placing additional stents, excluding the initial one. Major bleeding included intracranial, upper, and lower gastrointestinal hemorrhage. MACE covered postoperative AMI, acute heart failure, stroke, severe arrhythmia, and all-cause in-hospital mortality. AMI was diagnosed with serum enzyme elevation and symptoms. AHF involved sudden symptoms due to cardiac dysfunction. Severe arrhythmias included atrioventricular block, atrial flutter or fibrillation, and ventricular flutter or fibrillation. Data were from discharge summaries and postoperative records. Long-term endpoint was all-cause readmission rate, confirmed through medical records or communication. Follow-up averaged 1 year.

### Statistical analysis

In order to assess the impact of IPI levels on clinical outcomes in patients, we divided IPI into four groups based on quartiles. Enumeration data were expressed as numbers and percentage values (%), analyzed using the χ^2^ test.When χ^2^ test test conditions were not met, Fisher exact test was used, and values of *P* < 0.05 (two-sided) were considered significant. Shapiro–Wilk test was used to test the normality of continuous variables. Continuous variables were expressed as mean ± standard deviation (for normal distribution) and analyzed using ANOVA analysis, or as median and interquartile range (IQR) for non-normal distribution, the Mann–Whitney U test was used. First, univariate logistic regression analysis was used to determine the potential risk factors for in-hospital mortality (*P* < 0.05), and then multivariate logistic regression analysis was used to confirm that the previously significant variables were independent factors (*P* < 0.05). Multivariate Cox proportional hazard regression and Kaplan–Meier survival curve were used to evaluate all-cause readmission rate. All statistical analyses were performed using SPSS 26.0 software (https://www.ibm.com/products/spss-statistics). The graph was drawn using GraphPad Prism 9.3.1 software (https://www.graphpad.com/).

### Ethics approval

Ethical Approval was obtained from the Fujian Medical University Union Hospital ethics committee (2023KY032) prior to data collection.

### Informed consent

Informed consent was provided by all participants.

## Results

### Baseline characteristics

Among the 3,340 patients who underwent CAG and/or PCI, the average age was 63.96 ± 10.53 years, with 922 (27.6%) being female, and 1950 (58.4%) undergoing PCI. The mean IPI level in these patients was 0.74 [interquartile range: 0.11, 1.94]. Patients were stratified into four groups based on baseline IPI levels (Group 1, ≤ 0.11, n = 835; Group 2, 0.12–0.74, n = 835; Group 3, 0.75–1.93, n = 835; Group 4, ≥ 1.94, n = 835). Notably, higher IPI levels were associated with increased incidence of valvular heart disease (VHD) (41.7%,* p* < 0.001), heart failure (HF) (6.3%, *p* < 0.001), liver dysfunction (11.4%, *p* < 0.001), and chronic kidney disease (CKD) (16.6%, *p* < 0.001). Conversely, the incidence of hypertension (50.2%, *p* < 0.001), diabetes mellitus (DM) (27.4%, *p* < 0.001), hyperlipidemia (21.2%, *p* < 0.001), and acute coronary syndrome (ACS) (39.8%, *p* < 0.001) was lower in patients with higher IPI levels. Furthermore, patients in the highest IPI quartile had a higher prevalence of atrial fibrillation (AF) (23.5%, *p* < 0.001), pulmonary hypertension (17.8%,* p* < 0.001), and pulmonary infection (65.4%, *p* < 0.001), and tended to have longer hospital stays (*p* < 0.001). However, they had a lower history of myocardial infarction (2.3%, *p* < 0.001) and PCI (7.1%, *p* < 0.001). It is worth noting that with increasing IPI levels, patients' admission systolic and diastolic blood pressures, as well as left ventricular ejection fraction (LVEF), were negatively correlated (*p* < 0.001).

Patients in group 4 exhibited elevated preoperative creatinine levels (*p* < 0.001) and baseline urea nitrogen levels (*p* < 0.001). However, these patients also displayed a tendency toward reduced glomerular filtration rates (*p* < 0.001). Conversely, individuals with higher IPI levels tended to have elevated absolute neutrophil counts (*p* < 0.001) and white blood cell counts (WBC) (*p* < 0.001), but diminished absolute lymphocyte counts (*p* < 0.001). Moreover, the NLR was notably higher among subjects with IPI levels in the second and fourth quartiles (*p* < 0.001). Additionally, patients with IPI levels in the highest quartile exhibited decreased red blood cell count (RBC) levels (*p* < 0.001), concomitant with reductions in ALP (*p* < 0.001) and hemoglobin (Hb) levels (*p* < 0.001), while CRP (*p* < 0.001) and blood glucose (*p* < 0.001) levels increased.

Patients in group 4 had higher levels of preoperative creatinine (*p* < 0.001) and baseline urea nitrogen (*p* < 0.001), but these patients also tended to have lower glomerular filtration rate (*p* < 0.001). Individuals with higher IPI levels tended to have higher absolute neutrophil counts (*p* < 0.001) and white blood cell counts (WBC) (*p* < 0.001), but had lower absolute lymphocyte counts (*p* < 0.001). At the same time, NLR was higher in subjects with IPI levels in the second and fourth quartiles (*p* < 0.001). In addition, patients with IPI levels in the highest quartile had lower RBC levels (*p* < 0.001), accompanied by a decrease in ALP(*p* < 0.001), hemoglobin (Hb) (*p* < 0.001), while CRP (*p* < 0.001) and blood glucose (*p* < 0.001) levels increased.

In terms of medication, we observed that the use of diuretics increased in tandem with higher IPI levels. Conversely, the use of other medications such as calcium channel blockers, angiotensin converting enzyme inhibitors, angiotensin receptor blockers, angiotensin receptor enkephalinase inhibitors (ACEI/ARB/ARNI),β-blockers, dual antiplatelet therapy (DAPT), and statins exhibited an inverse relationship with IPI levels (*p* < 0.001). Regarding surgical characteristics, it is noteworthy that patients in the highest quartile of IPI levels had the highest number of infarcted arteries (*p* < 0.001). Conversely, patients with IPI levels in the lowest quartile were more likely to receive hydration therapy (*p* < 0.001) and undergo repeat angiography within seven days (*p* < 0.001), with a greater amount of contrast agent used. Furthermore, there was a significant negative correlation between IPI levels and the likelihood of undergoing PCI (80.4 vs 65.7 vs 49.2 vs 38.2%, *p* < 0.001), indicating that patients with lower IPI levels were more inclined to undergo PCI. More detailed baseline information is provided in Table 1.

**Table 1:** Baseline characteristics in patients undergoing CAG and/or PCI.

	Inflammatory Prognostic Index Quartile					
Variables	Quartile 1 (≤ 0.11)	Quartile 2 (0.12–0.74)		Quartile 3 (0.75–1.93)	Quartile 4 (≥ 1.94)	*P*-value
	(n = 835)	(n = 835)		(n = 835)	(n = 835)	
**Demographic and clinical characteristics**						
Age, years, M (SD)	63.49 ± 10.31	64.54 ± 10.92	63.77 ± 10.38		64.04 ± 10.50	0.209
Female sex*,* n (%)	200 (24.0)	235 (28.1)	576 (30.4)		622 (27.9)	0.029
Current smoking, n (%)	278 (33.3)	276 (33.1)	248 (29.7)		253 (30.3)	0.222
*Current drinking,* n (%)	599 (7.1)	58 (6.9)	51 (6.1)		66 (7.9)	0.509
SBP, mm Hg, M (SD)	131.80 ± 19.06	129.84 ± 19.62	127.22 ± 20.09		125.55 ± 21.10	< 0.001
DBP, mm Hg, M (SD)	79.28 ± 12.69	78.19 ± 12.42	78.08 ± 27.16		75.57 ± 13.54	< 0.001
BMI, kg/m^2^, M (SD)	24.22 ± 3.37	24.34 ± 2.98	23.85 ± 3.10		23.52 ± 3.24	< 0.001
LOS, days, M (SD)	7.88 ± 6.12	9.66 ± 7.09	12.29 ± 9.01		16.15 ± 10.97	< 0.001
EF (%), M (SD)	62.68 ± 9.74	59.41 ± 11.87	59.55 ± 12.63		57.32 ± 13.11	< 0.001
Prior MI, n (%)	105 (12.6)	58 (6.9)	27 (3.2)		19 (2.3)	< 0.001
Prior CVA, n (%)	55 (6.6)	52 (6.2)	40 (4.8)		59 (7.1)	0.245
Prior PCI, n (%)	20 (2.4)	20 (2.4)	10 (1.2)		13 (1.6)	0.174
Prior CABG, n (%)	215 (25.8)	141 (16.9)	96 (11.5)		59 (7.1)	< 0.001
**Complication**						
Hypertension*,* n (%)	533 (63.8)	492 (58.9)	430 (51.5)		419 (50.2)	< 0.001
DM*,* n (%)	320 (38.3)	276 (33.1)	231(27.7)		229 (27.4)	< 0.001
Hyperlipemia, n (%)	291 (34.9)	285 (34.1)	189 (22.6)		143 (17.1)	< 0.001
CKD, n (%)	53 (6.3)	65 (7.8)	86 (10.3)		139 (16.6)	< 0.001
Hepatic insufficiency, n (%)	39 (4.7)	60 (7.2)	65 (7.8)		95 (11.4)	< 0.001
ACS, n (%)	580 (69.5)	509 (61.0)	354 (42.4)		332 (39.8)	< 0.001
Pulmonary infection, n (%)	115 (13.8)	235 (28.1)	420 (50.3)		546 (65.4)	< 0.001
Valvular heart disease, n (%)	131 (15.7)	198 (23.7)	279 (33.4)		348 (41.7)	< 0.001
AF, n (%)	81 (9.7)	130 (15.6)	146 (17.5)		196 (23.5)	< 0.001
PAH, n (%)	26 (3.1)	64 (7.7)	130 (15.6)		149 (17.8)	< 0.001
HF, n (%)	17 (2.0)	34 (4.1)	23 (2.8)		53 (6.3)	< 0.001
**Medications before procedures**						
Diuretics, n (%)	139 (16.6)	250 (29.9)	342 (41.0)		452 (54.1)	< 0.001
CCB, n (%)	275 (32.9)	202 (24.2)	167 (20.0)		126 (15.1)	< 0.001
ACEI/ARB/ARNI, n (%)	326 (39.0)	266 (31.9)	211 (25.3)		189 (22.6)	< 0.001
β-blockers, n (%)	470 (56.3)	426 (51.0)	408 (48.9)		340 (40.7)	< 0.001
Statins, n (%)	715 (85.6)	588 (70.4)	469 (56.2)		370 (44.3)	< 0.001
DAPT, n (%)	725 (86.8)	589 (70.5)	444 (53.2)		353 (42.3)	< 0.001
**Procedural characteristics**						
PCI, n (%)	671 (80.4)	549(65.7)	411(49.2)		319(38.2)	< 0.001
Number of infarcted arteries	2.70 ± 0.79	2.75 ± 0.76	2.65 ± 0.83		2.75 ± 0.82	< 0.001
Stent number	1.51 ± 0.82	1.59 ± 0.86	1.62 ± 0.92		1.65 ± 0.95	0.094
Hydration therapy, n (%)	806 (96.5)	764 (91.5)	732 (87.7)		743 (89.0)	< 0.001
Repeated angiography(7 days)	689 (82.5)	586 (70.2)	463 (55.4)		367 (44.0)	< 0.001
Contrast volume, ml, M (SD)	347.97 ± 143.76	305.69 ± 165.52	301.45 ± 153.54		254.60 ± 174.38	< 0.001
**Baseline chemistry**						
ALP*,* g/L, M (SD)	40.99 ± 3.59	39.91 ± 3.74	39.35 ± 4.31		36.86 ± 5.38	< 0.001
UREA, mmol/L, M (SD)	5.74 ± 2.21	6.11 ± 3.19	6.39 ± 3.41		7.25 ± 4.54	< 0.001
Scr, μmol/L, M (SD)	80.29 ± 29.72	86.78 ± 48.50	91.83 ± 70.86		106.11 ± 104.56	< 0.001
Hemoglobin, g/L, M (SD)	137.01 ± 17.07	133.50 ± 17.66	132.33 ± 18.49		125.53 ± 22.98	< 0.001
NE, 10^9/L, M (SD)	3.86 ± 1.46	4.66 ± 2.19	4.62 ± 2.25		6.50 ± 3.52	< 0.001
WBC, 10^9/L, M (SD)	6.48 ± 1.83	7.30 ± 2.55	7.18 ± 2.52		8.77 ± 3.67	< 0.001
Lymphocyte, 10^9/L, M (SD)	1.97 ± 0.73	1.92 ± 0.95	1.85 ± 0.65		1.49 ± 0.64	< 0.001
RBC, 10^12/L, M (SD)	4.51 ± 0.58	4.43 ± 0.62	4.42 ± 0.65		4.23 ± 0.84	< 0.001
Platelet, 10^9/L, M (SD)	218.79 ± 57.53	222.87 ± 70.99	222.73 ± 70.25		224.73 ± 81.19	0.295
TC, mmol/L, M (SD)	4.19 ± 1.43	4.29 ± 1.13	4.33 ± 1.11		4.24 ± 1.19	0.126
GFR, mL/min, M (SD)	78.93 ± 23.57	75.33 ± 23.71	72.90 ± 22.78		71.63 ± 21.75	< 0.001
Blood glucose, mmol/L, M (SD)	5.97 ± 2.41	6.14 ± 2.50	5.91 ± 2.33		6.40 ± 3.10	0.002
NLR, MED (IQR)	1.93 (1.45,2.62)	2.35 (1.63, 3.33)	2.22 (1.73,3.18)		3.81 (2.61,6.72)	< 0.001
Hs-CRP, mg/L, MED (IQR)	0.80 (0.44, 1.28)	4.61 (2.97, 8.42)	24.35 (15.06,24.35)		43.00 (24.35,88.05)	< 0.001

*SBP* systolic blood pressure, *DBP* diastolic blood pressure, *BMI* body mass index, *LOS* length of stay, *EF* ejection fraction, *MI* myocardial infa*rction*, *CVA* cerebrovascular accident, *PCI* percutaneous coronary intervention, *CABG* coronary artery bypass graft surgery, *DM* diabetes mellitus, *CKD* chronic kidney disease, *ACS* acute coronary syndrome, *AF* atrial fibrillation, *PAH* pulmonary artery hypertension, *HF* heart failure, *CCB* Calcium Channel Blockers, *ACEI or ARB or ARNI* angiotensin-converting enzyme inhibitors or angiotensin receptor blockers or angiotensin receptor–neprilysin inhibitors, *DAPT dual* *antiplatelet* therapy, *ALP* albumin, *Scr* serum creatinine, *NE* neutrophils, *WBC* White blood cells, *RBC* red blood cells, *TC* total cholesterol, *GFR* glomeruar filtration rate, *NLR* neutrophil to lymphocyte ratio, *Hs-CRP* high-sensitivity C-reactive protein.

### IPI as a predictor of CIN risk and clinical outcome

**Figure 2:**
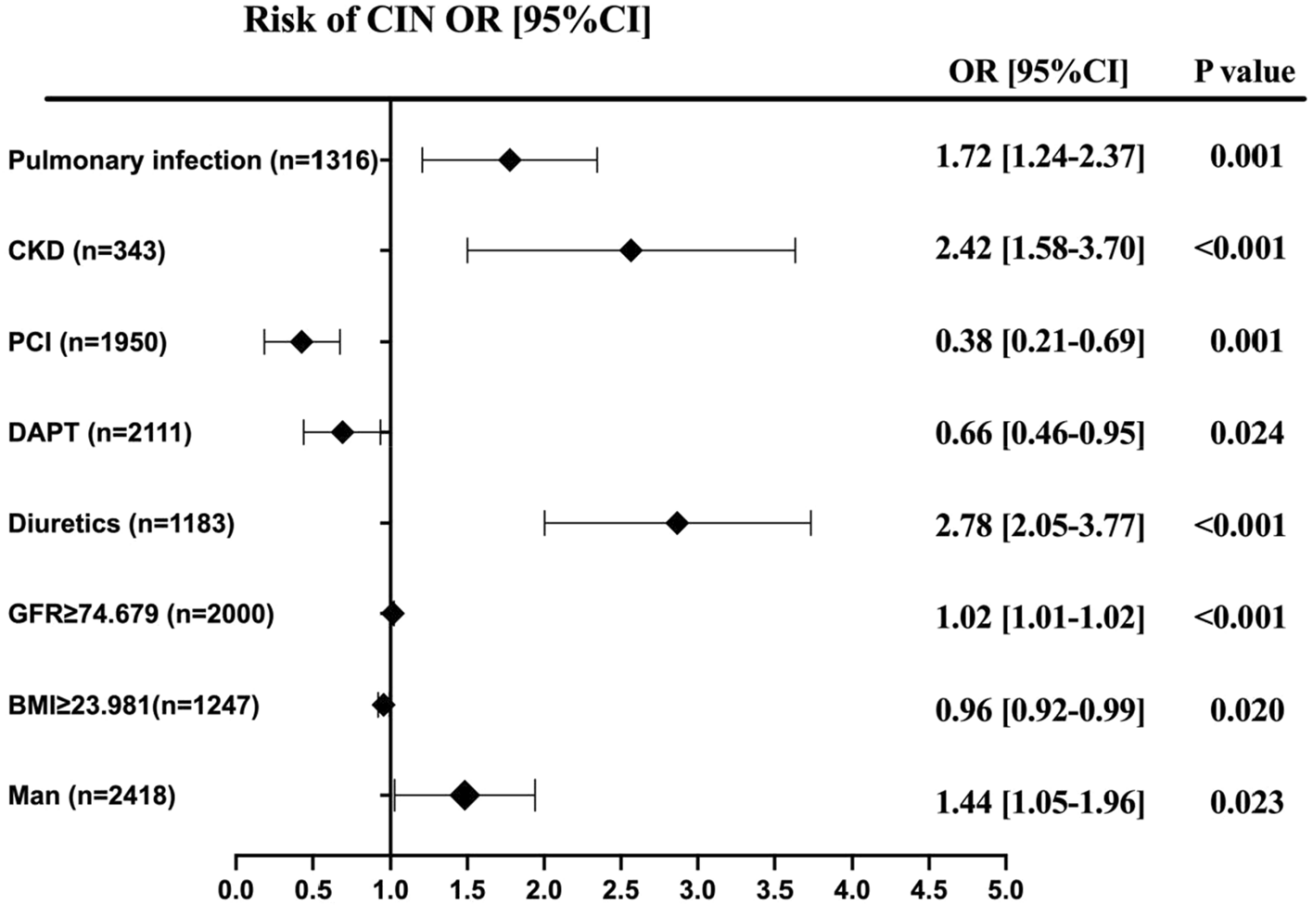
Predictors of CIN in patients undergoing CAG and/or PCI. Forest plot for the effects sizes of individual predictors of CIN in patients undergoing CAG and/or PCI. Abbreviations: *CKD,* chronic kidney disease; PCI, percutaneous coronary intervention; DAPT, *dual* *antiplatelet* therapy; GFR, glomeruar filtration rate; BMI, body mass index.

In the quartile analysis, the distribution of IPI levels was as follows: ≤ 0.11 (4.2%), 0.12–0.74 (9.7%), 0.75–1.93 (16.5%), and ≥ 1.94 (22.2%). These results reveal a strong positive correlation between high IPI levels and the risk of CIN. Even after adjusting for baseline confounders in model 2, this difference remained significant (*p* = 0.001). Notably, variables such as absolute neutrophil count, white blood cell count, absolute lymphocyte count, and hemoglobin were excluded from the model due to collinearity (variance inflation factor [VIF] > 5). Subgroup analysis indicated that male patients faced a 1.44 times higher risk of CIN compared to females. Moreover, PCI and DAPT were identified as protective factors against CIN (*p* < 0.05), while preoperative CKD, pulmonary infection, diuretic use, NYHA class III, and glomerular filtration rate emerged as independent risk factors (*p* < 0.05) (Fig. 2).

Furthermore, a significant positive correlation was observed between patients' IPI levels and all-cause in-hospital mortality (0.2% vs 1.1% vs 1.3% vs 3.4%, *p* < 0.001), with the highest quartile driving this association. However, multivariate logistic regression analysis failed to confirm this relationship (adjusted odds ratio [aOR] 2.50; 95% CI 0.49–12.78; *p* = 0.348). Additionally, there were no significant differences in the incidence of repeated revascularization and major bleeding across different IPI levels (*p* > 0.05). These conclusions were further supported by adjusted results.

**Figure 3:**
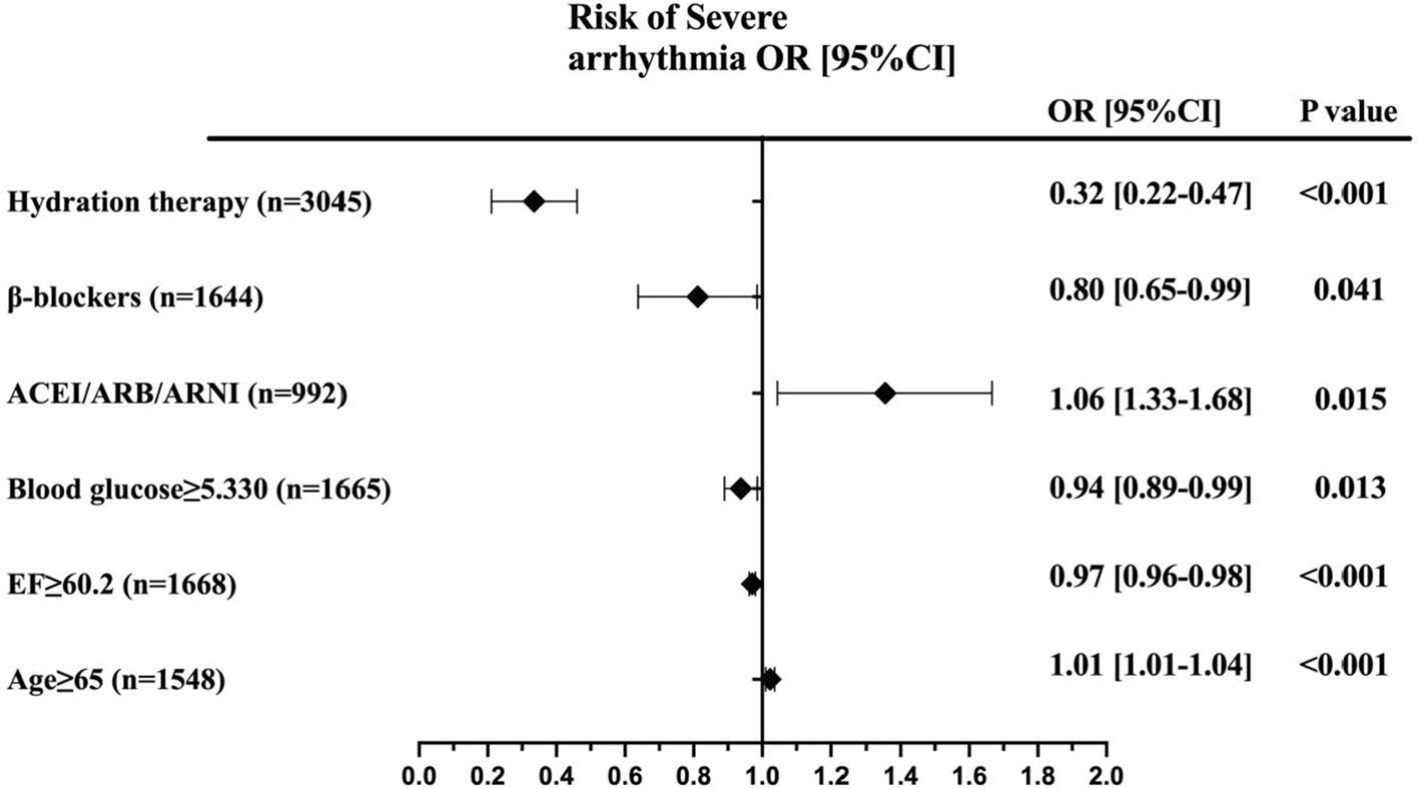
Predictors of acute severe arrhythmia in patients undergoing CAG and/or PCI. Forest plot for the effects sizes of individual predictors of severe arrhythmia in patients undergoing CAG and/or PCI. Abbreviations: ACEI or ARB or ARNI, angiotensin-converting enzyme inhibitors or angiotensin receptor blockers or angiotensin receptor–neprilysin inhibitors; EF, ejection fraction.

**Figure 4:**
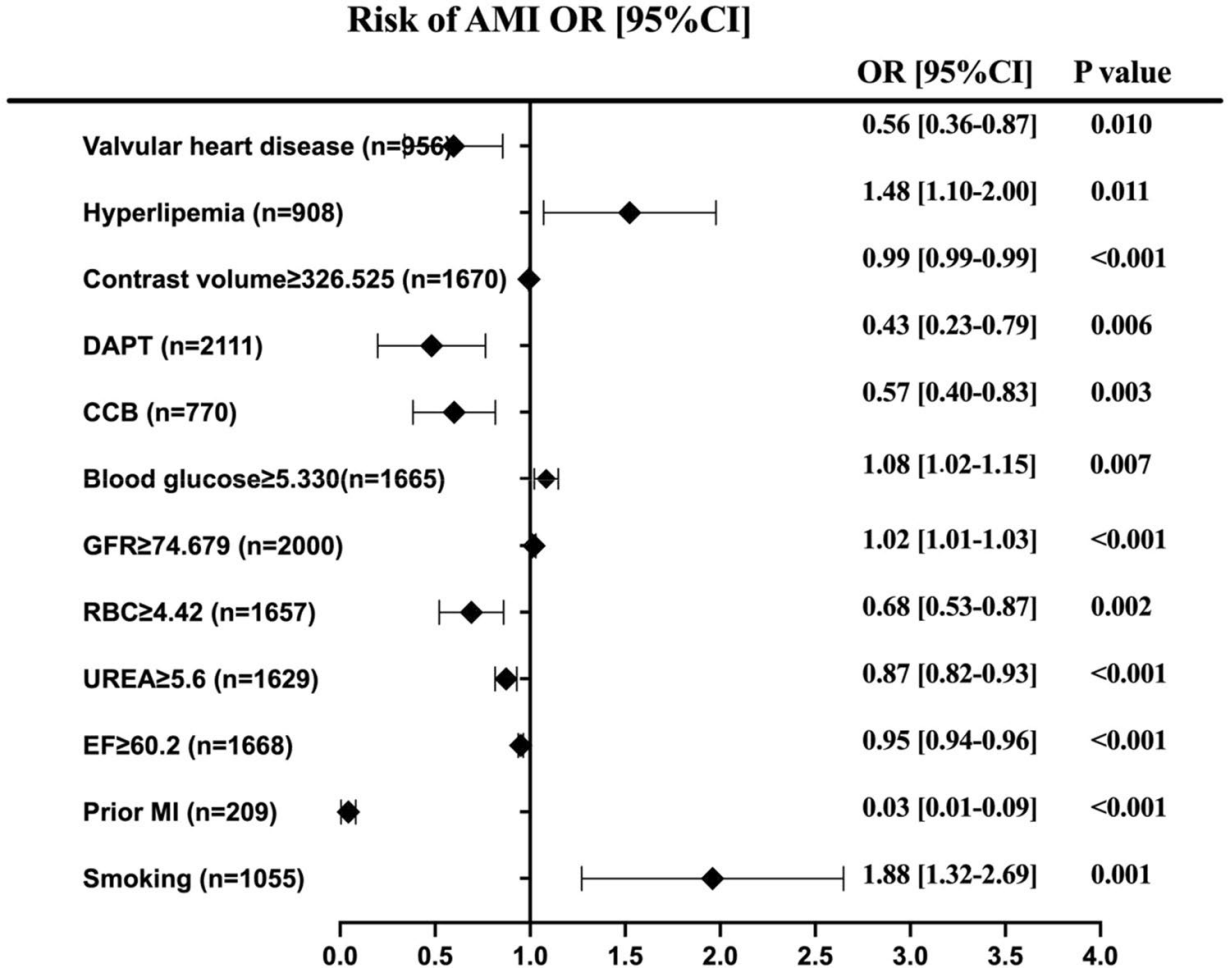
Predictors of acute myocardial infarction in patients undergoing CAG and/or PCI. Forest plot for the effects sizes of individual predictors of acute myocardial infarction in patients undergoing CAG and/or PCI. Abbreviations: DAPT, *dual* *antiplatelet* therapy; CCB, Calcium Channel Blockers; GFR, glomeruar filtration rate; RBC, red blood cells; EF, ejection fraction; MI, myocardial infa*rction.*

In addition, different baseline IPI levels were found to be independently associated with postoperative MACE, including arrhythmia and acute myocardial infarction (AMI), in patients undergoing CAG and/or PCI (*p* < 0.001). Specifically, the incidence of arrhythmia was highest among patients with baseline IPI levels in the highest quartile, although no significant difference was initially observed between the groups (3.2%, *p* > 0.05). However, subsequent multivariate analysis revealed significant differences between the groups (aOR 0.50; 95% CI 0.35–0.69; *p* < 0.001) (Fig. 3). Similarly, baseline IPI levels were independently associated with the risk of AMI in patients (p < 0.001), with this association further confirmed after adjusting for covariates (aOR 3.00; 95% CI 1.89–4.76; *p* < 0.001) (Fig. 4). Moreover, there was no significant difference observed between IPI levels and the risk of stroke (*p* > 0.05) (Table 2).

**Table 2:** The risk of CIN and clinical outcomes in patients undergoing CAG and/or PCI.

		Inflammatory Prognostic Index Quartile				
		Quartile 1 (≤ 0.11)	Quartile 2 (0.12–0.74)	Quartile 3 (0.75–1.93)	Quartile 4 (≥ 1.94)	*P*-value
**In-hospital outcomes**	Model	(n = 835)	(n = 835)	(n = 835)	(n = 835)	
**CIN**	CIN	35 (4.2)	81 (9.7)	138 (16.5)	185 (22.2)	< 0.001
	Non-CIN	800 (95.8)	754 (90.3)	697 (83.5)	650 (77.8)	NA
	Model 1	Reference	2.40 (1.59–3.62)	4.35 (2.96–6.40)	6.48 (4.44–9.45)	< 0.001
	Model 2	Reference	1.67 (1.06–2.64)	2.16 (1.39–3.35)	2.33 (1.50–3.64)	0.001
**Repeated revascularization**	Repeated revascularization	49(5.9)	37 (4.4)	40 (4.8)	30 (3.6)	0.172
	Non-Repeated revascularization	786(94.1)	798 (95.6)	795 (95.2)	805 (96.4)	NA
	Model 1	Reference	0.75 (0.48–1.17)	0.83 (0.54–1.27)	0.60 (0.38–0.96)	0.194
	Model 2	Reference	0.60 (0.36–1.00)	1.00 (0.59–1.70)	0.63 (0.33–1.20)	0.129
**Hematorrhea**	Hematorrhea	22 (2.6)	23 (2.8)	24 (2.9)	37 (4.4)	0.121
	Non-Hematorrhea	813 (97.4)	812 (97.2)	811 (97.1)	789 (95.6)	NA
	Model 1	Reference	1.03 (0.57–1.87)	1.11 (0.62–2.00)	1.71 (1.00–2.93)	0.129
	Model 2	Reference	1.01 (0.55–1.88)	0.84 (0.43–1.64)	1.08 (0.55–2.12)	0.873
***MACEs***						
AMI	AMI	138 (16.5)	262 (31.4)	188 (22.5)	269 (32.2)	< 0.001
	Non-AMI	697 (83.5)	573 (68.6)	647 (77.5)	566 (67.8)	NA
	Model 1	Reference	2.39 (1.89–3.03)	1.54 (1.20–1.96)	2.49 (1.97–3.15)	< 0.001
	Model 2	Reference	1.97 (1.37–2.83)	1.68 (1.12–2.52)	3.00 (1.89–4.76)	< 0.001
Stroke	*Stroke*	94 (11.3)	97 (11.6)	85 (10.2)	101 (12.1)	0.646
	Non-*Stroke*	741 (88.7)	738 (88.4)	750 (89.8)	734 (87.9)	NA
	Model 1	Reference	0.98 (0.72–1.34)	0.90 (0.65–1.23)	1.06 (0.78–1.44)	0.762
	Model 2	Reference	1.00 (0.72–1.40)	0.81 (0.56–1.16)	0.95 (0.64–1.40)	0.601
Severe arrhythmia	*Severe arrhythmia*	285 (34.1)	285 (34.1)	273 (32.7)	306 (36.6)	0.392
	Non-*Severe arrhythmia*	550 (65.9)	550 (65.9)	562 (67.3)	529 (63.4)	NA
	Model 1	Reference	0.96 (0.78–1.18)	0.92 (0.75–1.13)	1.09 (0.89–1.34)	0.378
	Model 2	Reference	0.73 (0.56–0.95)	0.61 (0.46–0.81)	0.50 (0.35–0.69)	< 0.001
*Acute heart failure*	*Acute heart failure*	1 (0.1)	2 (0.2)	5 (0.6)	16 (1.9)	< 0.001
	*Non-Acute heart failure*	834 (99.9)	833 (99.8)	830 (99.4)	819 (98.1)	NA
	Model 1	Reference	1.95 (0.18–21.55)	4.93 (0.57–42.33)	16.03 (2.12–121.20)	0.001
	Model 2	Reference	0.98 (0.08–12.24)	1.10 (0.10–12.33)	2.86 (0.28–29.05)	0.457
*All-cause in-hospital mortality*	All-cause in-hospital mortality	2 (0.2)	9 (1.1)	11 (1.3)	28 (3.4)	< 0.001
	Non-All-cause in-hospital mortality	833 (99.8)	826 (98.9)	824 (98.7)	807 (96.6)	NA
	Model 1	Reference	4.45 (0.96–20.69)	5.52 (1.22–24.98)	14.31 (3.40–60.28)	< 0.001
	Model 2	Reference	3.89 (0.76–20.04)	1.89 (0.35–10.35)	2.50 (0.49–12.78)	0.348
***Long-term outcome***						
*All-cause readmission*	*All*-*cause* *readmission*	387 (46.3)	323 (38.7)	209 (25.0)	207 (24.8)	< 0.001
	Non-*All*-*cause* *readmission*	448 (53.7)	512 (61.3)	626 (75.0)	628 (75.2)	NA
	Model 1	Reference	1.09 (0.92–1.30)	1.13 (0.93–1.38)	1.47 (1.21–1.79)	0.002
	Model 2	Reference	1.03 (0.85–1.25)	1.08 (0.86–1.36)	1.14 (0.88–1.46)	0.780

*CIN* contrast-induced nephropathy, *AMI* acute myocardial infarction, *MACE* major adverse cardiovascular events.

Model 1 was adjusted for age and sex.

Model 2 was adjusted for Model 1 plus other risk factors.

**Figure 5:**
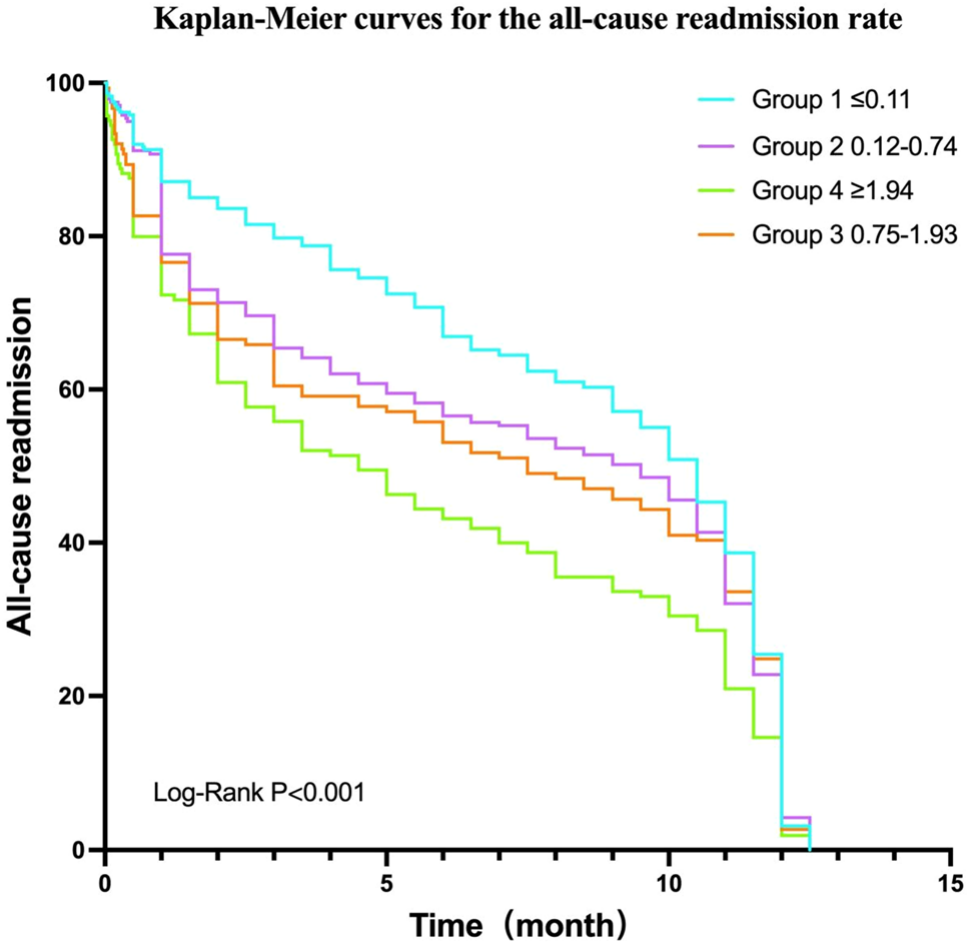
Kaplan–Meier curves for the all-cause readmission rate. Inflammatory prognostic index levels: Group 1, ≤ 0.11, n = 835; Group 2, 0.12–0.74, n = 835; Group 3, 0.75–1.93, n = 835; Group 4, ≥ 1.94, n = 835.

During the 1-year follow-up, 1095 out of 3340 patients were readmitted (32.8%). According to the results of univariate analysis, a higher baseline IPI level was associated with a lower rate of all-cause readmission (*p* < 0.001); however, multivariate analysis did not confirm this result. Kaplan–Meier curves for the all-cause readmission rate as shown below(Fig. 5)(Table 2).

## Discussion

This study is the first to examine the link between IPI levels and the risk of CIN and clinical outcomes in patients having CAG and/or PCI. Higher IPI levels were independently associated with increased CIN risk, and baseline IPI levels predicted severe arrhythmia and AMI. These findings suggest that IPI could be a useful prognostic marker in this patient population.

CIN is a complication that often arises in patients undergoing CAG and/or PCI, with notable morbidity and lethality^19^. Its development is likely associated with factors such as immunity, oxidative stress, and inflammatory responses^20^. While the exact pathophysiological mechanism remains uncertain, it is hypothesized to involve the cytotoxicity of contrast agents on renal tubules, leading to aggravated renal vasoconstriction, endothelial dysfunction, renal medulla ischemia, and hypoxia, ultimately resulting in renal tubular cell injury and death. Our analysis of 3340 patients undergoing CAG and/or PCI revealed a clear relationship between IPI levels and the risk of CIN. Particularly, patients in the highest quartile of IPI levels, indicative of more severe inflammatory responses, demonstrated a significantly elevated risk of CIN (*p* < 0.001). This association persisted even after adjusting for baseline confounders, consistent with prior studies^21^. Additionally, factors such as endothelial dysfunction, oxidative stress, and renal vasoconstriction have been identified as potential contributors to CIN^22^. Iodine contrast media (ICM) can induce activating transcription factor-2 (ATF2) to activate the oxidative stress system and make it participate in the inflammatory response. Moreover, ICM-induced renal parenchymal hypoxia can cause excessive release of reactive oxygen species (ROS), which directly leads to renal tubular and vascular endothelial dysfunction. Furthermore, subgroup analysis indicated that PCI treatment and preoperative use of DAPT were protective against CIN, whereas preoperative CKD and diuretic use increased the risk. These findings are in line with existing research^23,24^, suggesting that PCI may improve renal hemodynamics, while diuretic use may exacerbate renal tubular toxicity^25^. Identifying high-risk individuals is crucial for implementing targeted interventions.

An increasing body of research suggests that inflammation plays a crucial role in the development and destabilization of atherosclerotic plaques, highlighting its significance in CAD^26–28^. Our study, focusing on patients undergoing CAG and/or PCI, revealed a notable association between baseline levels of the IPI and postoperative MACE, including AMI and severe arrhythmia (*p* < 0.05). Specifically, patients in the highest quartile of IPI demonstrated the highest incidence of AMI, a finding that remained significant even after adjusting for baseline confounders (*p* < 0.001). Subsequent subgroup analysis identified hyperlipidemia and the extent of coronary artery involvement as independent risk factors for AMI, while preoperative DAPT emerged as a protective factor, consistent with previous studies^21^. The severity of the inflammatory response, as reflected by IPI levels, correlates with the magnitude of AMI, which is closely linked to immune-inflammatory responses and oxidative stress^29^. The intensity of the inflammatory response during AMI affects infarct size and subsequent ventricular remodeling^30^, serving as an indicator of AMI severity to some extent. Interestingly, while initially no difference in severe arrhythmia incidence was observed among patients with varying IPI levels (*p* > 0.05), this association became significant after adjustment (*p* < 0.001). Furthermore, subgroup analysis identified atrial fibrillation as an independent risk factor for severe arrhythmia in patients. However, existing research on the relationship between inflammation and arrhythmia risk presents conflicting conclusions^31,32^, likely due to variations in study populations and designs. Therefore, future studies with longer follow-up periods and larger, multicenter samples are needed to obtain more definitive evidence on the association between inflammatory factors and arrhythmias.

Coronary stent implantation is the primary method for revascularization in patients with CHD^19^. In-stent restenosis (ISR) is a significant postoperative complication that has attracted considerable attention. The pathogenesis of ISR is complex, involving vascular endothelial cell injury and apoptosis^38,39^, excessive proliferation and migration of vascular smooth muscle cells (VSMC), in-stent intimal hyperplasia, in-stent thrombosis, and persistent vascular inflammation. At present, there are also studies have shown that radioactive exposure such as ultra-high dose rate FLASH irradiation (FLASH-IR)stimulates endothelial cell activation, causes inflammatory response activation and thrombophilia, and induces and accelerates vascular atherosclerosis, which can also lead to ISR^43–45,47,49^. Previous studies have reported that 17% to 32% of patients with stent implantation develop ISR, typically occurring 6 to 12 months after PCI^33^. However, existing research predominantly focuses on the long-term association between individual inflammatory factor levels and ISR, overlooking the exploration of the relationship between IPI levels and repeated revascularization. Our study findings indicate no significant association between IPI and repeated revascularization (*p* > 0.05). This contrasts with some prior research conclusions^34^, possibly due to population differences in inflammatory responses and the duration of inflammation persistence. Further investigations are necessary to clarify these inconsistencies and comprehensively understand the role of inflammation in ISR and repeated revascularization.

After nearly 1 year of follow-up in this study, a significant correlation was observed between IPI and the all-cause readmission rate. However, after adjusting for covariates, Cox regression analysis did not provide further evidence to support this relationship (*p* > 0.05). The study findings indicated that the risk of all-cause readmission in male patients was 1.3 times higher than that in female patients (*p* > 0.05), consistent with findings from other studies^35^. This could be attributed to the higher mortality rate and incidence of adverse cardiac events among elderly male patients post-PCI compared to females^36^. Additionally, elderly male patients tend to have higher rates of smoking, drinking, and being overweight compared to females. Furthermore, research by domestic scholars has shown that elderly male patients exhibit lower compliance with chronic disease treatment and medication than elderly female patients^37^, leading to a higher risk of all-cause readmission in male patients. Additionally, we observed significantly lower all-cause readmission rates among patients who underwent PCI compared to those who did not (*p* < 0.001). This highlights the importance of focusing on patients who do not receive PCI treatment and ensuring timely PCI intervention based on the disease condition.

## Limitations

Our study is subject to limitations. It is a single-center retrospective study, and there may be biases (such as selection bias) present. Therefore, future research should aim for greater perfection and stricter quality control in order to minimize potential sources of error. Future research should prioritize multicenter prospective studies for broader insights. Also, we lack long-term follow-up data, focusing mainly on short-term in-hospital prognosis. Moreover, The occurrence and development of CIN involve many factors. This study is limited to the comparison of basic data such as age, gender, course of disease and laboratory biochemical examination, the changes of pathology and related molecular mechanisms have not been discussed, further research is needed in the future. Lastly, IPI levels may change during hospitalization, so future studies should monitor them dynamically. Despite limitations, our study is the largest to date and the first to explore IPI levels' association with patient prognosis in coronary angiography and/or percutaneous coronary intervention.

## Conclusion

This study is the first to investigate the IPI in patients undergoing CAG and/or PCI. We found that IPI is significantly associated with clinical prognosis, predicting the risk of CIN and postoperative outcomes independently. Its affordability and ease of use make it a valuable tool for early intervention strategies. Moving forward, more structured trials with increased participant involvement are needed to confirm our findings.

## Data Availability

The datasets used and analysed during the current study are available from the corresponding author upon reasonable request.
